# Fairness, trust, and digital inclusion in older adults’ intention to continue using community smart services in China

**DOI:** 10.3389/fpubh.2026.2004189

**Published:** 2026-10-30

**Authors:** Guanxi Chen, Jiajun Chen

**Affiliations:** 1 School of Design, NingboTech University, Ningbo, China; 2 Department of Business Administration, Joongbu University, Goyang, Republic of Korea

**Keywords:** community smart services, continuance intention, digital inclusion, older adults, platform trust, procedural fairness

## Abstract

**Introduction:**

Community smart services increasingly shape older adults’ access to public and health-related resources, making continued use an important dimension of digital inclusion. Drawing on the Theory of Planned Behavior (TPB), this study examines how public service fairness, digital self-efficacy, digital burden, perceived risk, and platform trust relate to older adults’ continuance intention.

**Methods:**

Data were obtained from 520 older adults with matched responses across three survey waves in Ningbo, Hangzhou, and Shaoxing, China. Partial least squares structural equation modeling (PLS-SEM) was used to examine the hypothesized relationships, supplemented by predictive analysis, necessary condition analysis (NCA), and follow-up interviews.

**Results:**

Attitude, subjective norm, and perceived behavioral control were positively associated with continuance intention. Platform trust showed a strong positive association with attitude. Perceived risk was negatively associated with perceived behavioral control, although its specific indirect association with continuance intention was not statistically significant. NCA revealed moderate ceiling relationships for the three TPB constructs.

**Discussion:**

The findings suggest that older adults’ continued engagement with community smart services depends on more than technological access. Trustworthy and understandable service procedures, perceived control, and accessible support may contribute to sustained use. These findings underscore the importance of age-friendly service design, transparent and correctable procedures, capability support, responsive assistance, and reliable offline alternatives in promoting digital inclusion.

## Introduction

Community smart services now support routine public transactions, health-related information, and community participation through digital channels (1). For older adults, difficulty navigating these channels can restrict access to everyday resources relevant to healthy aging. Although digital provision can expand service coverage, limited capability, insufficient support, and procedural friction may make repeated use difficult (2, 3). Continued engagement therefore raises questions about the fairness, manageability, and reliability of digitally mediated public services, beyond the availability of technology alone.

Research on technology acceptance, digital inclusion, and digital government offers three perspectives on these questions. Public-service fairness concerns understandable procedures and inclusive routes to services (4, 5). Digital capability and administrative burden concern users’ confidence and the demands of repeated tasks (6, 7). Trust and risk concern provider dependability and possible adverse consequences (8, 9). How these experiences relate to continued-use intentions within a common behavioral framework merits examination.

The Theory of Planned Behavior (TPB) distinguishes attitude (ATT), subjective norm (SN), and perceived behavioral control (PBC) as proximal correlates of intention (10). These judgments provide a framework for examining older adults’ evaluations of community smart services, perceived encouragement from others, and confidence in managing future use.

The study considers procedural fairness (PF), accessibility fairness (AF), digital self-efficacy (DSE), digital burden (DB), platform trust (TR), and perceived risk (PR) within the three perspectives above. Their conceptual distinctions and connections to the TPB constructs are developed in the literature review.

Using a three-wave survey of older community-service users in Zhejiang, we examine associations between these six contextual perceptions, the three TPB constructs, and continuance intention (CI).

PLS-SEM provides the main structural and indirect-association analysis. Predictive modelling, necessary-condition analysis, and follow-up interviews offer complementary perspectives on prediction, possible thresholds, and everyday service experience.

The study extends the application of TPB to digitally mediated public services by integrating fairness, task-related capability and burden, and trust–risk judgments. It also clarifies how these contextual perceptions show differing associations with older adults’ evaluations, social expectations, and perceived control, informing research on digital inclusion after initial adoption.

## Literature review and hypotheses

### Older adults’ continuance use of community smart services

Community smart services differ from commercial applications because service delivery is embedded in public rules and local governance relationships (4). For older users, usable access depends on both digital skills and the availability of dependable assistance (11, 12). Research on older adults’ digital public-service use highlights the roles of subjective skills, institutional support, and accessible service channels (2, 5).

Technology acceptance models, including TAM and UTAUT, offer established explanations of digital service adoption through usefulness, ease of use, social influence, and facilitating conditions (13–15). Continued use also involves judgments grounded in service experience, including whether repeated transactions remain worthwhile, manageable, and dependable (16). Research on later-life computer and health-application use likewise associates ongoing engagement with accumulated experience and capability beliefs (17, 18).

The first research strand concerns public-service fairness and inclusion. Procedural justice foregrounds consistent rules and opportunities for correction; digital inclusion foregrounds service routes accessible to people with differing capabilities (11, 19, 20).

The second strand links digital capability to administrative burden. Digital-divide research examines skill and confidence differences (21, 22), while public-administration research identifies learning and compliance costs created by complex procedures (6, 23, 24). Digital burden captures the user-experienced demands of those procedures.

The third strand concerns trust and risk in digital public services. Institutional reliability, responsible problem handling, privacy, and potential service errors are prominent in digital-government research (9, 25, 26). Trust concerns expectations of the provider, whereas perceived risk concerns possible adverse outcomes for the user.

Together, these strands motivate six specific antecedents: PF and AF for procedures and access; DSE and DB for task capability and demands; and TR and PR for provider expectations and potential consequences. Rather than using service quality as a broad composite, the model examines these service experiences in relation to the distinct TPB judgments of ATT, SN, and PBC.

### TPB as an integrative framework for continuance intention

TPB distinguishes evaluations of a behavior (ATT), perceived social expectations (SN), and judgments of behavioral feasibility (PBC) as related components of intention (10). Their relative importance depends on the social and practical demands of the context (27).

For older adults, community smart-service use takes place within family and neighborhood support networks, making subjective norm relevant alongside individual service evaluations (28, 29). A favorable attitude may also coexist with doubts about completing verification steps or correcting errors; PBC captures this practical dimension of repeated use (2, 30). TPB therefore offers a way to examine evaluative, social, and control-related judgments together in post-adoption service use (18).

The six contextual perceptions may be associated with more than one TPB component: fair procedures and inclusive routes can inform evaluations and social endorsement; capability and burden relate particularly to perceived feasibility; and trust and risk concern expectations about reliability and consequences. The TPB framework accommodates these overlapping associations without treating the antecedents as interchangeable (27, 31).

The hypotheses focus on relationships with the clearest theoretical grounding, while the full structural model also examines additional associations between the contextual antecedents and the three TPB constructs.

### Public-service fairness and inclusion

Procedural justice in public services concerns understandable decisions, consistent standards, and opportunities for correction or appeal (4, 19, 20). These features can inform favorable evaluations and reduce uncertainty about completing service transactions (32).

The present study distinguishes two aspects of fairness in community service provision. Procedural fairness (PF) concerns consistent and understandable rules, transparent decisions, and opportunities to request correction or review (20). Accessibility fairness (AF) concerns older adults’ perceptions of the availability of service channels, assistance when difficulties arise, and offline alternatives that keep community services accessible across differences in digital capability (33). These arrangements share practical features with the facilitating conditions considered in technology-adoption research, especially the availability of assistance and support. In this public-service setting, AF focuses on inclusive access to the service, including ways to obtain it without completing a digital transaction independently. Perceived behavioral control (PBC), by comparison, concerns an individual’s judgment about their ability and control over continuing to use the digital service. The distinction is thus between arrangements for obtaining a service and perceived control over performing the target behavior (2, 11, 34).

Transparent, correctable procedures may support favorable evaluations and judgments of feasible use (23, 35). Accessible channels and alternative ways to obtain the same service may be valued without necessarily increasing confidence in independent digital completion (2, 11). Comparing PF and AF across ATT and PBC captures these differences, while social endorsement remains relevant to older adults’ service choices (36).

The fairness-related hypotheses are:

H1: Procedural fairness (PF) is positively associated with attitude (ATT).

H2: Accessibility fairness (AF) is positively associated with attitude (ATT).

H3: Procedural fairness (PF) is positively associated with perceived behavioral control (PBC).

H4: Accessibility fairness (AF) is positively associated with perceived behavioral control (PBC).

### Digital capability and administrative burden

Digital inclusion includes differences in capability and the demands imposed by digital tasks (21, 22). For community service users, learning unfamiliar functions occurs alongside repeated verification, information entry, and error resolution (6, 23, 24). DSE captures confidence in completing tasks, whereas DB captures the experienced costs of these institutionally designed procedures.

Higher DSE may accompany persistence with unfamiliar functions and greater confidence in completing future transactions (30, 37). This belief is especially pertinent to PBC, while potentially relating to service evaluations and perceived encouragement.

Digital burden includes time, effort, and uncertainty associated with multistep procedures and repeated verification (6, 24). These demands may accompany lower perceived control; repeated difficulties may also shape evaluations and recommendations from family or community staff (2, 38).

The capability- and burden-related hypotheses are:

H5: Digital self-efficacy (DSE) is positively associated with perceived behavioral control (PBC).

H6: Digital burden (DB) is negatively associated with perceived behavioral control (PBC).

### Trust and risk judgments in digital public services

Perceived risk (PR) concerns anticipated privacy exposure, mistakes, information loss, or difficulties reversing an incorrect action. Unlike platform trust, which concerns provider dependability, risk can persist even when a service provider is generally trusted. Such concerns may relate to less favorable evaluations and lower perceived control, and can intersect with advice from family or community staff (39–42).

The risk-related hypotheses are:

H7: Perceived risk (PR) is negatively associated with attitude (ATT).

H8: Perceived risk (PR) is negatively associated with perceived behavioral control (PBC).

Platform trust (TR) concerns expectations of service reliability, information protection, and responsible problem handling (43). Digital-government studies associate provider dependability with citizens’ willingness to engage with services involving personal information (9, 25, 26).

Trust may accompany more favorable attitudes and stronger perceived control when users expect dependable service and support (25, 40, 44). Endorsement by relatives and community staff may similarly depend on perceived institutional reliability (45).

The trust-related hypotheses are:

H9: Platform trust (TR) is positively associated with attitude (ATT).

H10: Platform trust (TR) is positively associated with perceived behavioral control (PBC).

### Core TPB pathways to continuance intention

Continuance intention (CI) refers to willingness to choose a service again after prior use. In community smart services, the decision may depend on whether the channel remains worthwhile, socially endorsed, and feasible to use (10, 27).

ATT concerns the value of continued use (46); SN concerns encouragement from relatives, peers, and community contacts (28, 47); and PBC concerns perceived ability and resources for completing future tasks despite service difficulties (48). Each represents a distinct aspect of an older user’s judgment about continued service use.

A positive service evaluation need not coincide with confidence in completing future tasks, and encouragement need not eliminate perceived constraints. These three judgments are therefore examined together in relation to CI. The corresponding hypotheses are:

H11: Attitude (ATT) is positively associated with continuance intention (CI).

H12: Subjective norm (SN) is positively associated with continuance intention (CI).

H13: Perceived behavioral control (PBC) is positively associated with continuance intention (CI).

### Indirect associations through the TPB components

The model also estimates 18 specific indirect associations between the six contextual perceptions and continuance intention through ATT, SN, and PBC. These estimates distinguish the statistical associations carried by the three TPB components for each antecedent.

For each antecedent, indirect associations are estimated through ATT, SN, and PBC separately, yielding 18 specific indirect paths. These estimates are examined in the mediation analysis. Figure 1 brings these relationships together.

**Figure 1:**
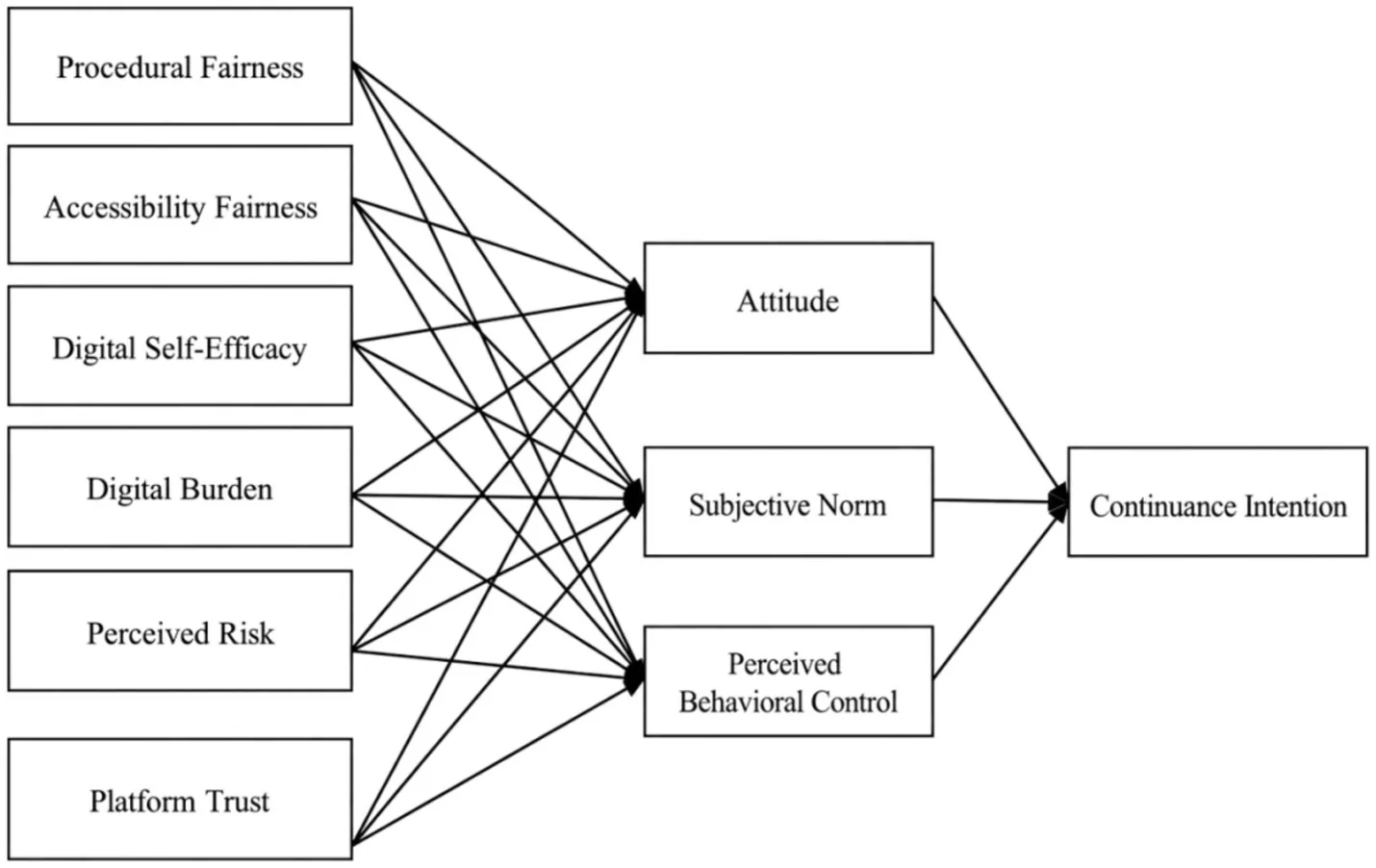
Theoretical framework of continuance intention.

## Materials and methods

### Case

Ningbo, Hangzhou, and Shaoxing were selected as varied settings of community digital-service provision in Zhejiang Province. Ningbo has an established community-level service integration context (49), while Hangzhou and Shaoxing have followed differing smart-city development pathways (50). These sites provided opportunities to examine older adults’ experiences with community smart services in different local settings.

The study considered everyday community services including appointment registration, repair and grievance requests, utility payments, information inquiries, health follow-up reminders, and community activity registration. Across these functions, service access may involve both digital procedures and offline assistance, making them relevant to fairness, task demands, and trust judgments.

### Research method

This study used a three-wave time-lagged survey of older adults who had experience using community smart services in Ningbo, Hangzhou, and Shaoxing, China. The survey separated measurement of contextual perceptions at T1, the three TPB constructs at T2, and continuance intention at T3, following the conceptual sequence of the model. Each construct was assessed at one wave.

The survey covered eight communities in Zhejiang Province, China: three in Ningbo, three in Hangzhou, and two in Shaoxing. Communities with established smart-service provision were purposively selected, and eligible older residents were recruited on site through convenience sampling without predefined numerical quotas. Participants were eligible if they had used at least one community smart service in the preceding 30 days, could independently understand and complete the questionnaire. Services included appointment booking, repair requests, utility payments, information inquiries, activity registration, and health follow-up reminders.

Trained research assistants administered paper questionnaires at the community sites. The source measures were reviewed by two language professionals for conceptual clarity and cross-language consistency before adaptation into Chinese. The final Chinese wording was checked for readability in the community-service context. Participants received standardised instructions about voluntary participation, anonymous responses, and independent completion, with clarification of survey procedures available when necessary.

To link the three survey waves anonymously, each participant received a paper card with a randomly assigned code at T1 and was asked to retain it through T3. Completed questionnaires were sealed in envelopes and deposited in collection boxes before retrieval and data entry. Responses were linked using these codes without attaching personally identifying information to the analytical dataset. Screening considered the completeness of core measurement responses as well as apparent inconsistencies or irregular response patterns. The analyses used complete three-wave matched cases (*N* = 520).

Following the third survey wave, 12 older adults with experience using community smart services participated in semi-structured interviews. Interview recruitment was organised separately from the anonymously coded survey: individuals interested in a follow-up conversation could volunteer through a separate contact and consent process, without linking contact details to their survey responses. Purposive selection considered age, gender, service-use frequency, and reliance on assistance. Interviews were conducted by trained members of the research team, lasted approximately 15–20 min, and explored recent service encounters, procedural clarity, available assistance, digital burden, risk, platform reliability, and future use intentions. With participants’ consent, interviews were audio-recorded and transcribed verbatim. Identifying details were removed from the transcripts before analysis. Trained research assistants independently coded the transcripts, beginning with categories informed by the study constructs while allowing additional themes to emerge from the accounts. Coding differences were discussed within the research team with reference to the transcripts until a shared interpretation was reached. Both recurrent and contrasting accounts were retained in the thematic synthesis; interview evidence was analysed independently of participants’ survey scores.

Ethical approval was obtained before data collection from the Ethics Committee. Before each survey wave, participants received information about the study purpose, survey procedures, voluntary participation, and confidentiality. Completing and returning a questionnaire indicated informed consent to participate in that survey wave. Participants could decline to participate or discontinue the questionnaire before returning it. Anonymous participant codes were used to link responses across waves, and no personally identifying information was linked to the analytical dataset.

#### Scale design

The Chinese-language survey comprised three wave-specific questionnaires covering 10 reflective constructs, each measured by three items. Six contextual constructs (18 items) were assessed at T1, the three TPB constructs (nine items) at T2, and continuance intention (three items) at T3. Participants rated the items on a five-point Likert scale ranging from 1 (strongly disagree) to 5 (strongly agree). The measures were adapted from established instruments for familiar community-service tasks. Expert review and pilot feedback informed refinements to item wording and contextual relevance. Table 1 summarises the measurement domains and principal sources; measurement quality is reported in Tables 2, 3.

**Table 1:** Measurement items and primary sources.

Construct (wave)	Item code	English questionnaire item	Primary sources
PF (T1)	PF1	The rules and steps for handling matters through community smart services are explained clearly.	Colquitt (53)
	PF2	The rules are generally applied consistently when handling the same type of community matter.	
	PF3	If I have questions about a service outcome, community smart services provide a way to ask for clarification or request a correction.	
AF (T1)	AF1	Community smart services provide service access points that are easy for older adults to find.	Kebede et al. (11); Giest and Samuels (23)
	AF2	When I have difficulty using online services, the community provides assistance from staff.	
	AF3	Even without using a mobile phone or the internet, I can handle relevant community matters through offline channels.	
DSE (T1)	DSE1	I am confident that I can learn unfamiliar steps involved in using community smart services.	Compeau and Higgins (54); Eastin and LaRose (55)
	DSE2	I am confident that I can complete the required steps when handling routine community matters by mobile phone or online.	
	DSE3	When I encounter a simple problem while using these services, I believe I can find a solution.	
DB (T1)	DB1	I find the steps required to handle community matters through smart services troublesome.	Herd and Moynihan (56); Peeters (24)
	DB2	To complete a community service task, I often spend a considerable amount of time entering and checking information.	
	DB3	Repeated identity verification or resubmission of materials during the process feels burdensome to me.	
PR (T1)	PR1	When using community smart services, I worry that my personal information might be leaked.	Featherman and Pavlou (57); Bélanger and Carter (58)
	PR2	When handling community matters online, I worry that an operating mistake could have negative consequences.	
	PR3	If I enter or submit incorrect information, I worry that it may be difficult to change or withdraw it afterward.	
TR (T1)	TR1	I trust community smart services to provide relevant services consistently and reliably.	McKnight et al. (59); Bélanger and Carter (58)
	TR2	I trust community smart services to protect and handle my personal information appropriately.	
	TR3	When problems arise during the service process, I trust the responsible service provider to deal with them carefully.	
ATT (T2)	ATT1	I think continuing to use community smart services in the future is a good thing.	Ajzen (60); Bosnjak et al. (10)
	ATT2	For me, continuing to use community smart services in the future is worthwhile.	
	ATT3	Overall, I have a positive attitude toward continuing to use community smart services in the future.	
SN (T2)	SN1	My family wants me to continue using community smart services in the future.	Ajzen (60); Bosnjak et al. (10)
	SN2	Friends or neighbors I know would support me in continuing to use community smart services.	
	SN3	Community staff would encourage me to continue using community smart services.	
PBC (T2)	PBC1	When I need to handle community matters in the future, I believe I will be able to continue using the relevant smart services.	Ajzen (60); Bosnjak et al. (10)
	PBC2	I feel that whether I continue using community smart services in the future is mainly up to me.	
	PBC3	Even if I encounter some difficulties while using them, I still believe I can continue using community smart services.	
CI (T3)	CI1	I intend to continue using community smart services in the future.	Bhattacherjee (61)
	CI2	The next time I need to handle a relevant community matter, I will still choose to use community smart services.	
	CI3	Over the coming period, I plan to continue handling relevant community matters through community smart services.	

**Table 2:** Reliability and convergent validity.

Latent variable	Observed variable	Factor loading	Cronbach‘s *α*	CR	AVE
AF	AF1	0.837	0.800	0.882	0.714
	AF2	0.860			
	AF3	0.838			
ATT	ATT1	0.882	0.849	0.909	0.768
	ATT2	0.882			
	ATT3	0.864			
CI	CI1	0.883	0.860	0.914	0.781
	CI2	0.871			
	CI3	0.896			
DB	DB1	0.835	0.823	0.894	0.738
	DB2	0.864			
	DB3	0.878			
DSE	DSE1	0.888	0.836	0.901	0.753
	DSE2	0.828			
	DSE3	0.886			
PBC	PBC1	0.882	0.840	0.903	0.757
	PBC2	0.874			
	PBC3	0.854			
PF	PF1	0.866	0.794	0.879	0.707
	PF2	0.823			
	PF3	0.833			
PR	PR1	0.853	0.823	0.894	0.738
	PR2	0.850			
	PR3	0.873			
SN	SN1	0.850	0.836	0.901	0.753
	SN2	0.872			
	SN3	0.881			
TR	TR1	0.851	0.824	0.895	0.740
	TR2	0.872			
	TR3	0.857			

**Table 3:** Discriminant validity assessed using the heterotrait–monotrait ratio (HTMT).

Construct	AF	ATT	CI	DB	DSE	PBC	PF	PR	SN
ATT	0.365								
CI	0.050	0.532							
DB	0.130	0.270	0.045						
DSE	0.160	0.388	0.052	0.325					
PBC	0.169	0.586	0.482	0.393	0.430				
PF	0.271	0.372	0.039	0.041	0.155	0.246			
PR	0.177	0.203	0.032	0.360	0.165	0.256	0.092		
SN	0.319	0.574	0.492	0.270	0.354	0.492	0.319	0.229	
TR	0.300	0.597	0.060	0.269	0.271	0.326	0.222	0.241	0.452

Digital self-efficacy (DSE) concerns confidence in performing digital tasks, whereas perceived behavioral control (PBC) concerns control over the specific behavior of continuing to use a service. Procedural fairness (PF) concerns understandable rules and opportunities for correction, while platform trust (TR) concerns expectations of provider dependability. Accessibility fairness (AF) reflects perceived opportunities to obtain community services through accessible channels, assistance, or offline alternatives. It therefore captures an aspect of inclusive service provision, whereas PBC concerns the perceived feasibility of continued digital use.

### Data collection

Data were collected in three waves between March and August 2026. At T1 (March–April), 735 participants completed measures of procedural fairness, accessibility fairness, digital self-efficacy, digital burden, perceived risk, platform trust, and demographic characteristics. At T2 (May–June), 680 participants completed measures of attitude, subjective norm, and perceived behavioral control. At T3 (July–August), 627 participants completed the continuance intention survey. The number of participants decreased by 55 between T1 and T2 and by a further 53 between T2 and T3, corresponding to a cumulative follow-up attrition rate of 14.69%. Of the 627 records assessed at final screening, 520 complete three-wave matched cases were retained (82.93%). The final sample included 200 participants from Ningbo, 192 from Hangzhou, and 128 from Shaoxing. The numbers initially approached and declining participation were not systematically recorded during community recruitment. Available records did not provide a detailed breakdown of reasons for follow-up non-completion, and separate counts by exclusion reason were not compiled for the 107 records not retained after screening.

Of the 520 participants, 53.08% were women and 90.00% were aged 60–75 years. Most respondents were retired (80.00%), while 50.00% reported occasional trial use of community smart services. Table 4 reports the full demographic profile, service-use patterns, assistance requirements, and city distribution.

**Table 4:** Sample characteristics.

Category	Option	Frequency	%
Age	60–64 years	182	35.00%
	65–69 years	156	30.00%
	70–75 years	130	25.00%
	>75 years	52	10.00%
Gender	Male	244	46.92%
	Female	276	53.08%
Employment status	Still employed (including re-employed retirees)	104	20.00%
	Retired	416	80.00%
Living arrangement	Living with spouse	312	60.00%
	Living with children	156	30.00%
	Living alone	52	10.00%
Community smart service usage level	Occasional trial use	260	50.00%
	Habitual use	182	35.00%
	Stable reliance	78	15.00%
Assistance received	Frequently require assistance from others	52	10.00%
	Occasionally require assistance	208	40.00%
	Basically able to complete independently	260	50.00%
Study city	Ningbo	200	38.46%
	Hangzhou	192	36.92%
	Shaoxing	128	24.62%
Total sample size		520	

### Analytical strategy

The analytical strategy followed the study’s theoretical sequence. Partial least squares structural equation modeling was used to evaluate the measurement model, structural paths, and indirect effects. Predictive analysis and necessary condition analysis were then used as supplementary checks on predictive patterning and possible bottleneck conditions. Follow-up interviews were used to place the quantitative findings in the context of older adults’ actual service experiences.

#### Primary analysis: PLS-SEM

PLS-SEM was conducted in SmartPLS 4.1 using standardized estimates and the path-weighting scheme. The 10 constructs were specified reflectively with three indicators each. Reliability, convergent validity, HTMT discriminant validity, and structural paths were assessed. The TPB-based model (M1) estimated paths from the six contextual antecedents to ATT, SN, and PBC, followed by three paths to continuance intention. An extended model (M2) additionally included direct paths from PF, AF, DSE, DB, PR, and TR to CI. M1 percentile 95% bootstrap confidence intervals were obtained through an independent Python implementation of the same reflective PLS path-weighting specification, using the 520 matched indicator records and 10,000 case-resampling iterations (random seed 20261013). Both measurement weights and structural paths were re-estimated in each resample, and full-sample coefficients were checked against the SmartPLS estimates. For M2, inference used 10,000 bootstrap resamples and two-sided 95% percentile confidence intervals. Direct, specific indirect, aggregate indirect, and total associations were distinguished. The original SmartPLS *p*-values were retained for Benjamini–Hochberg false discovery rate (BH-FDR) adjustment across the 21 M1 structural paths and 18 specific indirect paths (39 tests), with adjusted *q*-values reported alongside *p*-values.

#### Supplementary predictive analysis

The supplementary predictive analysis used nine construct-level inputs (PF, AF, DSE, DB, PR, TR, ATT, SN, and PBC), each scored as the mean of its three items, to predict the three-item mean for continuance intention (CI). A feedforward multilayer perceptron (MLP) was estimated in Python using scikit-learn, with one hidden layer of 12 neurons, ReLU activation, and Adam optimisation (learning rate = 0.001; L2 penalty = 0.01; maximum 2,000 iterations). Early stopping reserved 15% of each training partition for validation, with a tolerance of 0.0001 and 40 iterations without improvement. Performance was assessed across 10 repeated random 70:30 training–test splits (random seeds 101–110). For each split, predictor and outcome standardisation was fitted using training observations only; RMSE was calculated after inverse transformation to the numerical response coding in the analysed item data. A ridge regression model (alpha = 1.0), evaluated on the same partitions, provided a linear benchmark. Permutation importance was measured as the increase in held-out mean squared error when each input was permuted 20 times per split. Average importance was normalised relative to the largest average (100%). Model settings were held constant across runs, and held-out test observations were not used for parameter fitting or tuning.

#### Supplementary necessary-condition analysis

Necessary condition analysis (NCA) was applied to the 520 matched observations using standardized construct scores exported from SmartPLS. For each of the nine candidate conditions, CE-FDH was used to estimate the non-decreasing ceiling envelope, and CR-FDH fitted a regression ceiling through its upper-left peer points. The ceiling zone was divided by the empirical scope of each condition–outcome pair to obtain effect size *d*. Significance was assessed through 1,999 permutations (fixed random seed: 20261013). The interpretation considered effect size, ceiling accuracy, and permutation results together; effects of 0.10–0.30 were treated as moderate.

#### Supplementary interview-based interpretation

Follow-up interviews provided examples of how older adults encountered procedural clarity, assistance, digital demands, risk, and platform reliability in everyday service use. The thematic findings are reported alongside the quantitative analyses to give context to the observed associations.

## Results

### Data analysis and hypothesis testing

We tested the hypotheses using partial least squares structural equation modeling (PLS-SEM) in SmartPLS 4.1.

#### Measurement model assessment

##### Reliability and convergent validity

The 10 reflective constructs showed satisfactory internal consistency and convergent validity (Table 2). Cronbach’s alpha ranged from 0.794 to 0.860, composite reliability from 0.879 to 0.914, and AVE from 0.707 to 0.781; all indicator loadings exceeded 0.80.

##### Discriminant validity

Discriminant validity was assessed using the heterotrait–monotrait ratio of correlations (HTMT). As shown in Table 3, all 45 HTMT values were below the conservative threshold of 0.85; the highest was between platform trust and attitude (0.597). For construct pairs of particular conceptual relevance, HTMT was 0.430 for DSE–PBC, 0.222 for PF–TR, and 0.169 for AF–PBC. These results support empirical discriminant validity across the measured constructs.

##### Common method Bias

Anonymous administration, standardized instructions, and temporal separation of measurements across three survey waves were adopted to reduce common method concerns. Outer and inner variance inflation factors (VIFs) were examined to assess indicator-level and structural-predictor collinearity, respectively, rather than as direct tests of common method bias.

### Structural model evaluation

The M1 structural estimates, comprising theory-focused and exploratory paths, are illustrated in Figure 2; numerical results and BH-FDR adjustments are presented in Table 5.

**Figure 2:**
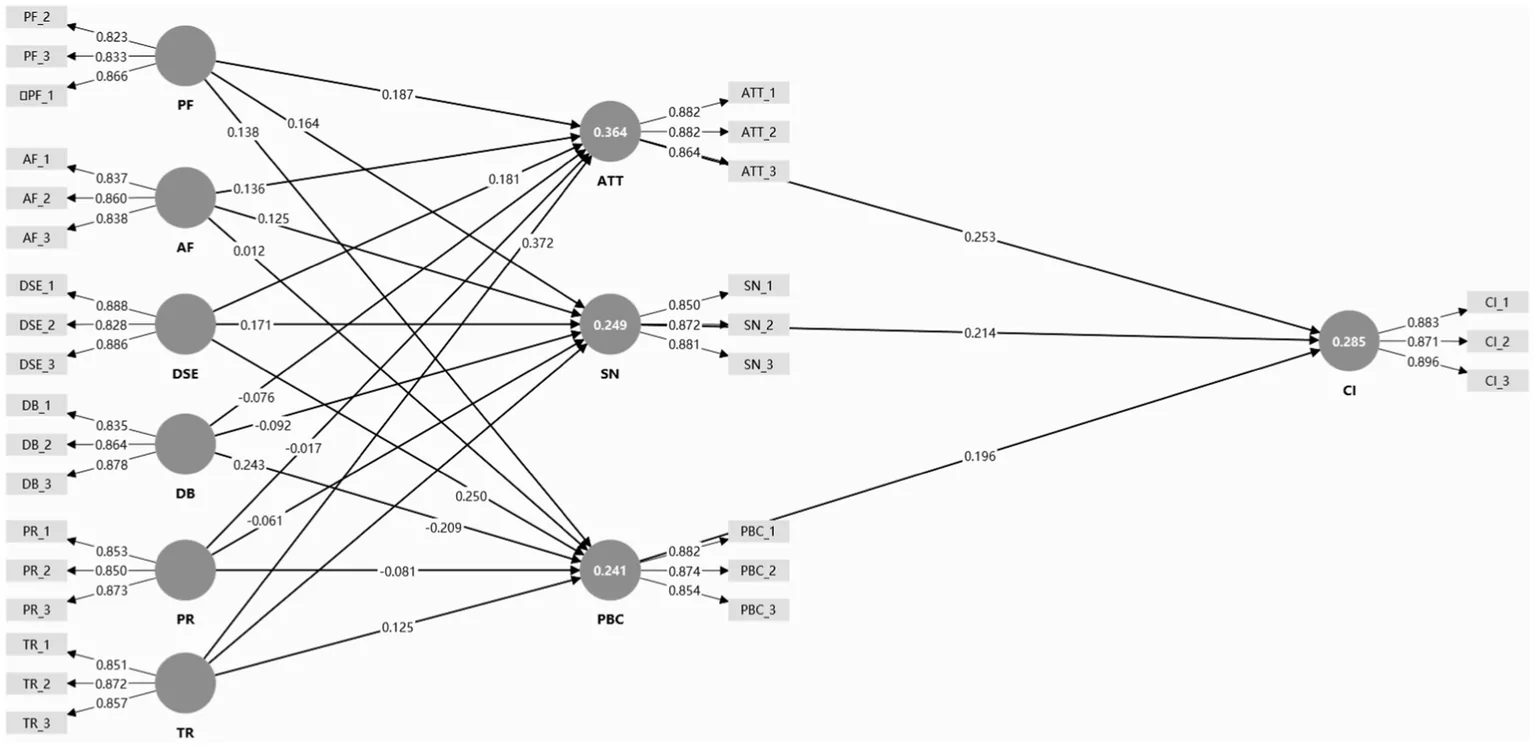
Full PLS-SEM structural model results (M1). The diagram presents the measurement indicators and all 21 estimated structural paths. Detailed indicator loadings are reported in Table 2, while standardized path coefficients and BH-FDR-adjusted significance levels are provided in Table 5.

**Table 5:** M1 structural paths: coefficients, significance tests, and 95% bootstrap confidence intervals.

Structural path	*β*	SE	*t*	*p*	BH-FDR *q*	95% CI
AF → ATT (H2)	0.136	0.035	3.929	<0.001	<0.002	[0.068, 0.203]
AF → PBC (H4)	0.012	0.041	0.295	0.768	0.774	[−0.068, 0.091]
AF → SN	0.125	0.038	3.318	0.001	≤0.002	[0.050, 0.199]
ATT → CI (H11)	0.253	0.046	5.556	<0.001	<0.002	[0.164, 0.345]
DB → ATT	−0.076	0.042	1.833	0.067	0.084	[−0.156, 0.001]
DB → PBC (H6)	−0.209	0.043	4.905	<0.001	<0.002	[−0.287, −0.129]
DB → SN	−0.092	0.041	2.238	0.026	0.036	[−0.172, −0.011]
DSE → ATT	0.181	0.038	4.771	<0.001	<0.002	[0.105, 0.255]
DSE → PBC (H5)	0.250	0.038	6.531	<0.001	<0.002	[0.176, 0.323]
DSE → SN	0.171	0.041	4.146	<0.001	<0.002	[0.090, 0.249]
PBC → CI (H13)	0.196	0.047	4.171	<0.001	<0.002	[0.107, 0.287]
PF → ATT (H1)	0.187	0.038	4.948	<0.001	<0.002	[0.114, 0.261]
PF → PBC (H3)	0.138	0.038	3.677	<0.001	<0.002	[0.061, 0.216]
PF → SN	0.164	0.039	4.228	<0.001	<0.002	[0.089, 0.239]
PR → ATT (H7)	−0.017	0.037	0.454	0.650	0.685	[−0.090, 0.056]
PR → PBC (H8)	−0.081	0.038	2.139	0.033	0.044	[−0.162, −0.004]
PR → SN	−0.061	0.039	1.573	0.116	0.133	[−0.141, 0.021]
SN → CI (H12)	0.214	0.045	4.739	<0.001	<0.002	[0.128, 0.301]
TR → ATT (H9)	0.372	0.036	10.256	<0.001	<0.002	[0.296, 0.443]
TR → PBC (H10)	0.125	0.040	3.107	0.002	0.003	[0.047, 0.205]
TR → SN	0.243	0.042	5.730	<0.001	<0.002	[0.159, 0.324]

#### Model fit evaluation

The structural model explained 36.4% of the variance in ATT, 24.9% in SN, 24.1% in PBC, and 28.5% in CI. SRMR was 0.040 for the saturated model and 0.072 for the estimated model (51). *R*^2^ values describe explanatory power, whereas SRMR provides a separate approximate-fit diagnostic.

### Path analysis

All 21 M1 structural paths were estimated. Table 5 reports the standardized coefficients, original *p*-values, BH-FDR-adjusted *q*-values, and 95% bootstrap confidence intervals for the 13 theory-focused and eight exploratory associations.

For public-service fairness, procedural fairness (PF) was positively associated with attitude (*β* = 0.187, *p* < 0.001; H1) and perceived behavioral control (*β* = 0.138, *p* < 0.001; H3). Accessibility fairness (AF) was positively associated with attitude (*β* = 0.136, *p* < 0.001; H2), but its association with PBC was not significant (*β* = 0.012, *p* = 0.768; H4). PF and AF were also positively associated with subjective norm (*β* = 0.164, *p* < 0.001; *β* = 0.125, *p* = 0.001, respectively). Overall, procedural fairness showed a broader pattern of association than accessibility fairness across the three TPB components.

Digital self-efficacy was positively associated with PBC (*β* = 0.250, *p* < 0.001; H5), whereas digital burden was negatively associated with PBC (*β* = −0.209, *p* < 0.001; H6). DSE was also positively associated with ATT (*β* = 0.181, *p* < 0.001) and SN (*β* = 0.171, *p* < 0.001). DB was negatively associated with SN (*β* = −0.092, *p* = 0.026; BH-FDR *q* = 0.036), but its association with ATT was not statistically significant (*β* = −0.076, *p* = 0.067; *q* = 0.084). These results point to perceived control as a particularly salient correlate of both digital capability and service-related burden.

Perceived risk was not significantly associated with ATT (*β* = −0.017, *p* = 0.650; H7), but showed a small negative association with PBC (*β* = −0.081, *p* = 0.033; *q* = 0.044; H8). Its association with SN was not statistically significant (*β* = −0.061, *p* = 0.116). The specific indirect associations of perceived risk with CI did not reach the BH-FDR-adjusted significance threshold.

Platform trust was positively associated with ATT (*β* = 0.372, *p* < 0.001; H9), PBC (*β* = 0.125, *p* = 0.002; H10), and SN (*β* = 0.243, *p* < 0.001). All three TPB components were positively associated with continuance intention: ATT → CI (*β* = 0.253, *p* < 0.001; H11), SN → CI (*β* = 0.214, *p* < 0.001; H12), and PBC → CI (*β* = 0.196, *p* < 0.001; H13). These associations remained significant after BH-FDR adjustment.

Of the 39 M1 structural and specific indirect tests, 29 met the BH-FDR criterion (*q* < 0.05). The AF–PBC and PR–ATT paths and the three risk-related indirect associations did not meet that criterion.

#### Mediation effects

Specific indirect associations were estimated for all six contextual antecedents through each of the three TPB components (Table 6). Unadjusted *p*-values, BH-FDR-adjusted *q*-values, and 95% percentile bootstrap confidence intervals are reported for all 18 paths.

**Table 6:** M1 specific indirect associations: estimates and 95% bootstrap confidence intervals.

Indirect path	*β*	SE	*t*	*p*	BH-FDR *q*	95% CI
AF → ATT → CI	0.034	0.011	3.251	0.001	≤0.002	[0.015, 0.058]
DSE → PBC → CI	0.049	0.014	3.570	<0.001	<0.002	[0.025, 0.076]
PR → SN → CI	−0.013	0.009	1.414	0.158	0.176	[−0.033, 0.004]
PF → PBC → CI	0.027	0.010	2.789	0.005	0.008	[0.010, 0.048]
TR → SN → CI	0.052	0.013	3.951	<0.001	<0.002	[0.028, 0.080]
DB → ATT → CI	−0.019	0.011	1.741	0.082	0.097	[−0.043, 0.000]
PR → PBC → CI	−0.016	0.009	1.804	0.072	0.088	[−0.036, −0.001]
DSE → ATT → CI	0.046	0.012	3.795	<0.001	<0.002	[0.024, 0.073]
TR → PBC → CI	0.025	0.010	2.583	0.010	0.014	[0.008, 0.044]
PF → ATT → CI	0.047	0.012	4.001	<0.001	<0.002	[0.026, 0.073]
PR → ATT → CI	−0.004	0.009	0.458	0.647	0.685	[−0.024, 0.014]
TR → ATT → CI	0.094	0.019	4.837	<0.001	<0.002	[0.058, 0.134]
AF → SN → CI	0.027	0.010	2.693	0.007	0.011	[0.010, 0.048]
AF → PBC → CI	0.002	0.008	0.287	0.774	0.774	[−0.014, 0.018]
DB → SN → CI	−0.020	0.010	1.958	0.051	0.066	[−0.041, −0.002]
DSE → SN → CI	0.037	0.011	3.443	0.001	≤0.002	[0.017, 0.060]
DB → PBC → CI	−0.041	0.013	3.178	0.002	0.003	[−0.069, −0.019]
PF → SN → CI	0.035	0.011	3.181	0.002	0.003	[0.017, 0.058]

The results showed that several statistically significant specific indirect associations involved attitude (ATT), subjective norm (SN), and perceived behavioral control (PBC). First, within the fairness → CI chain, procedural fairness showed a significant positive indirect association with continuance intention through ATT (PF → ATT → CI: *β* = 0.047, *p* < 0.001), SN (PF → SN → CI: *β* = 0.035, *p* = 0.002), and PBC (PF → PBC → CI: *β* = 0.027, *p* = 0.005). Accessibility fairness also showed a significant indirect association through ATT (AF → ATT → CI: *β* = 0.034, *p* = 0.001) and SN (AF → SN → CI: *β* = 0.027, *p* = 0.007), whereas the pathway through PBC was not significant (AF → PBC → CI: *β* = 0.002, *p* = 0.774), which is consistent with the direct-effect results.

Second, within the capability → CI chain, digital self-efficacy showed significant positive indirect associations through all three TPB constructs: DSE → ATT → CI (*β* = 0.046, *p* < 0.001), DSE → SN → CI (*β* = 0.037, *p* = 0.001), and DSE → PBC → CI (*β* = 0.049, *p* < 0.001). These findings suggest that stronger capability beliefs among older adults may accompany continuance intention simultaneously through value evaluation, perceived social support, and a stronger sense of control.

Third, within the platform trust → CI chain, the specific indirect associations of platform trust were also significant, with the attitudinal pathway being the strongest: TR → ATT → CI (*β* = 0.094, *p* < 0.001). The corresponding indirect associations through SN (TR → SN → CI: *β* = 0.052, *p* < 0.001) and PBC (TR → PBC → CI: *β* = 0.025, *p* = 0.010) were likewise significant.

Digital burden and perceived risk showed more differentiated indirect patterns. Digital burden had a negative indirect association with CI through PBC (DB → PBC → CI: *β* = −0.041, *p* = 0.002, *q* = 0.003), while its paths through ATT and SN did not meet the BH-FDR criterion (*q* = 0.097 and 0.066, respectively). For perceived risk, none of the three specific indirect paths met that criterion (all *q* > 0.05). The DB → SN → CI and PR → PBC → CI percentile confidence intervals were below zero, but their adjusted *q*-values were 0.066 and 0.088; these two estimates are therefore interpreted as inconclusive under the multiple-testing criterion.

For each antecedent, the aggregate indirect association in M1 is the sum of the three specific indirect estimates through ATT, SN, and PBC. Direct antecedent-to-CI paths are examined separately in M2.

Together, the indirect estimates indicate that platform trust was most strongly associated with CI through ATT, whereas the negative indirect association of digital burden was concentrated in the PBC pathway. The three TPB components therefore capture different aspects of the relationship between service experience and continuance intention.

#### Supplementary analyses

##### Extended-model sensitivity analysis (M2)

The extended model (M2) retained the TPB pathways and added direct associations between the six contextual antecedents and CI. Several direct and aggregate indirect coefficients had opposite signs, underscoring the relevance of model specification to their interpretation. Table 7 reports direct, aggregate indirect, and total associations, with 95% bootstrap confidence intervals for the direct and aggregate indirect estimates.

**Table 7:** Extended model (M2): standardized direct, aggregate indirect, and total associations with CI.

Antecedent	Direct *β*	95% CI direct	Aggregate indirect *β*	95% CI indirect	Total β*
PF	−0.227	[−0.298, −0.159]	0.182	[0.120, 0.249]	−0.045
AF	−0.096	[−0.166, −0.025]	0.110	[0.051, 0.168]	0.014
DSE	−0.197	[−0.268, −0.121]	0.213	[0.146, 0.282]	0.016
DB	0.106	[0.033, 0.179]	−0.125	[−0.195, −0.058]	−0.019
PR	0.039	[−0.025, 0.107]	−0.052	[−0.115, 0.007]	−0.012
TR	−0.251	[−0.328, −0.177]	0.293	[0.225, 0.363]	0.041

Total *β* is the sum of the direct and aggregate indirect coefficients; intervals are 95% percentile bootstrap CIs (10,000 resamples).

In M2, all three proximal TPB associations with CI remained positive: ATT → CI (*β* = 0.471, 95% CI [0.384, 0.558]), SN → CI (*β* = 0.342, 95% CI [0.263, 0.422]), and PBC → CI (*β* = 0.278, 95% CI [0.194, 0.364]). Among the specific indirect associations, TR → ATT → CI (*β* = 0.175, 95% CI [0.128, 0.226]) and DB → PBC → CI (*β* = −0.058, 95% CI [−0.092, −0.031]) illustrate the positive trust-related and negative burden-related patterns. These selected intervals complement the aggregate results in Table 7.

The ANN and ridge benchmark were evaluated across 10 repeated random 70:30 splits (364 training and 156 held-out observations per split). Mean ANN training RMSE was 0.546 (SD = 0.036), and held-out RMSE was 0.604 (SD = 0.030), compared with 0.563 (SD = 0.021) for ridge regression on the same held-out observations. Thus, the ANN did not improve upon the linear benchmark.

Permutation importance was greatest for ATT (100.0%), SN (71.7%), and PBC (53.8%). Among the six contextual antecedents, TR (36.0%), PF (26.0%), and DSE (20.9%) ranked highest, followed by AF (8.1%), DB (3.9%), and PR (2.6%; Table 8).

**Table 8:** Predictive importance of all nine ANN inputs: mean increase in held-out MSE and normalised importance (largest mean = 100%).

Input	Mean ΔMSE	Normalised importance	Overall rank
ATT	0.1682	100.0%	1
SN	0.1205	71.7%	2
PBC	0.0905	53.8%	3
TR	0.0606	36.0%	4
PF	0.0437	26.0%	5
DSE	0.0352	20.9%	6
AF	0.0136	8.1%	7
DB	0.0066	3.9%	8
PR	0.0044	2.6%	9

Mean importance reflects the increase in held-out mean squared error when each input was permuted; values were normalised to the largest mean importance (100%). The importance ranking and its underlying mean ΔMSE values are shown in Table 8.

For the six contextual antecedents, descending absolute aggregate indirect coefficients in M1 were TR (+0.1707), DSE (+0.1316), PF (+0.1094), DB (−0.0798), AF (+0.0636), and PR (−0.0334). ANN importance ranked them TR, PF, DSE, AF, DB, and PR. Indirect rankings use absolute magnitude (signs retained), whereas ANN importance indicates predictive sensitivity.

##### Necessary-condition check

The NCA identified moderate, statistically supported necessity effects for ATT (CE-FDH *d* = 0.1995), SN (*d* = 0.1654), and PBC (*d* = 0.1553), all *p* < 0.001. The corresponding CR-FDH results were consistent (*d* = 0.1616, 0.1420, and 0.1409, respectively). Table 9 reports the full CE-FDH and CR-FDH estimates, including ceiling-zone areas, empirical scopes, effect sizes, and permutation *p*-values.

**Table 9:** Necessary-condition results for continuance intention using CE-FDH and CR-FDH (*N* = 520).

Condition	Method	Accuracy (%)	Ceiling zone	Scope	Effect size (*d*)	*p*-value
AF	CE-FDH	100.00	2.0625	30.06	0.0686	0.4720
	CR-FDH	99.42	1.6917	30.06	0.0563	0.4080
ATT	CE-FDH	100.00	5.8178	29.16	0.1995	<0.001
	CR-FDH	98.85	4.7125	29.16	0.1616	<0.001
DB	CE-FDH	100.00	1.9723	29.63	0.0666	0.5695
	CR-FDH	99.62	1.8204	29.63	0.0614	0.4005
DSE	CE-FDH	100.00	3.2604	29.97	0.1088	0.1190
	CR-FDH	98.46	2.7542	29.97	0.0919	0.1340
PBC	CE-FDH	100.00	4.6197	29.75	0.1553	<0.001
	CR-FDH	99.42	4.1914	29.75	0.1409	<0.001
PF	CE-FDH	100.00	0.0224	28.08	0.0008	0.9795
	CR-FDH	100.00	0.0112	28.08	0.0004	0.9795
PR	CE-FDH	100.00	0.6010	29.81	0.0202	0.9485
	CR-FDH	100.00	0.3005	29.81	0.0101	0.9525
SN	CE-FDH	100.00	4.8523	29.33	0.1654	<0.001
	CR-FDH	98.65	4.1661	29.33	0.1420	<0.001
TR	CE-FDH	100.00	1.4892	27.41	0.0543	0.3395
	CR-FDH	98.85	1.6784	27.41	0.0612	0.2105

The six contextual antecedents did not show comparable statistically supported necessity evidence; for example, DSE had a CE-FDH effect size of 0.1088 (*p* = 0.119). These ceiling relationships represent a different statistical property from structural associations and are interpreted accordingly.

##### Follow-up interview findings: Interview themes

The interviews describe how older adults navigated routine digital services, including occasions when an apparently accessible service remained difficult to complete independently. The account below distinguishes recurrent experiences from differences across participants.

Themes were organised around participants’ accounts of service procedures, practical assistance, effort, uncertainty, and confidence in continued use. The findings include contrasting views within these themes. Quotations are attributed to anonymised interview identifiers, which are separate from the survey matching codes.

Interviewees described both the convenience of digital services and the practical reservations that arose when tasks became difficult or outcomes were uncertain.

Clear explanations and opportunities to correct errors featured in accounts of procedural fairness. One participant explained: “As long as the process is clearly explained and I can find someone to ask when something goes wrong, I feel this is something I can keep using.” (A06) The account reflects the value placed on understandable procedures and accessible problem resolution.

Accounts of assistance were less uniform. Some participants appreciated help from community staff or family members, clear service entry points, and the availability of offline alternatives. Others distinguished receiving help on one occasion from feeling able to complete the next task without assistance. As one participant observed, “someone helping me get in does not mean I will be able to use it by myself next time.” (A03) These views suggest that support and perceived independent control need not coincide.

Participants who were comfortable with routine smartphone tasks described trying unfamiliar steps and troubleshooting minor problems before requesting help. Expressions such as “I can learn it” and “I can handle it” reflected task-related confidence, although participants did not necessarily expect to complete every procedure alone.

Lengthy procedures, repeated verification, and uncertainty about correcting errors appeared in accounts of digital burden and perceived risk. One participant remarked: “It’s not that the service is bad; it’s just too troublesome to use, and I’m afraid that if I tap the wrong thing, it will become even more troublesome.” (A12) Another expressed concern that “if something goes wrong, I would not know what to do.” (A10) These accounts distinguish the effort required by a task from concern about the consequences of making a mistake, even when the service itself is considered useful.

Trust was often described in practical terms, particularly whether the digital channel was recognisably connected to community services and whether someone would take responsibility for problems. As one participant stated: “As long as I know this really belongs to the community and that someone will deal with it if there is a problem, I’m much less afraid of continuing to use it.” (A02) This reflects reassurance associated with an identifiable provider and a route for resolving difficulties.

Taken together, the interview accounts provide context for understanding how assistance, procedural clarity, digital demands, and confidence can coexist in older adults’ service experiences.

## Discussion

### TPB components and continuance intention

Attitude (ATT), subjective norm (SN), and perceived behavioral control (PBC) were positively associated with continuance intention (CI), consistent with the central propositions of the Theory of Planned Behavior (27). These findings suggest that older adults’ willingness to continue using community smart services is related not only to their evaluations of the services but also to perceived social expectations and confidence in managing future transactions.

The association of SN with CI was similar in magnitude to that of ATT, highlighting the relevance of social relationships in community-based digital services. Family members, neighbors, and community staff often provide encouragement and practical guidance, making continued engagement partly embedded in everyday social interactions (12). PBC captures a complementary dimension, reflecting whether older adults perceive subsequent digital transactions as manageable when unfamiliar procedures or errors arise. Together, these findings extend the application of TPB to post-adoption decisions in digitally mediated public services, where service evaluations, social expectations, and practical control beliefs are closely intertwined.

### Procedural fairness and accessibility fairness

Procedural fairness (PF) was positively associated with all three TPB components, whereas accessibility fairness (AF) was associated with ATT and SN but not PBC. This difference is particularly relevant to the distinction between fairness in service procedures and inclusive opportunities to obtain services. Clear rules, consistent decisions, and opportunities for correction may be associated with more favorable evaluations and greater confidence in navigating future transactions. By comparison, accessible channels, available assistance, and offline alternatives concern whether services remain obtainable across differences in digital capability.

This distinction also connects with research on older adults’ digital inclusion and public-service accessibility (34). Although AF shares practical features with facilitating conditions, its emphasis lies in inclusive service provision rather than individual confidence in completing digital tasks. The observed associations suggest that favorable perceptions of service accessibility do not necessarily coincide with stronger perceived control over independent digital use. Understanding how different forms of assistance relate to service access and capability development remains an important direction for further investigation.

### Digital capability and administrative burden

Digital self-efficacy (DSE) was positively associated with ATT, SN, and PBC, with its strongest association observed for PBC. This pattern is consistent with research highlighting the importance of task-related confidence in older adults’ digital engagement (30). DSE reflects confidence in learning and performing digital tasks, whereas PBC concerns perceived control over the specific behavior of continuing service use. Their relationship therefore connects general digital capability beliefs with judgments about future participation.

Digital burden (DB), in contrast, showed negative associations with PBC and SN, while its association with ATT was not statistically significant. Its negative indirect association with CI was most clearly reflected in the PBC pathway. These findings draw attention to the user-experienced demands of digital public services. Repeated verification, complex procedures, and uncertain error correction can make subsequent transactions appear difficult even when users recognize the value of a service. This interpretation connects the administrative burden literature, which emphasizes demands arising from institutional procedures, with older adults’ perceived capacity to manage digital tasks (23, 24).

### Platform trust and perceived risk

Platform trust (TR) and perceived risk (PR) displayed different patterns of association within the TPB framework. TR was positively associated with ATT, SN, and PBC, suggesting that expectations of reliable service provision, responsible information handling, and problem resolution are relevant to several aspects of older adults’ service judgments. This finding is consistent with research emphasizing institutional reliability in digital government and older adults’ engagement with digital services (9, 31).

PR, by contrast, showed a small negative association with PBC, while its associations with ATT and SN were not statistically significant. None of its three specific indirect associations with CI met the BH-FDR-adjusted significance criterion. Concerns about errors, privacy, and potentially adverse consequences were therefore most clearly reflected in perceived control over digital tasks. The supplementary predictive analysis also assigned PR the lowest held-out importance among the nine inputs.

These findings highlight a distinction between expectations of provider dependability and concerns about possible consequences for the user. Trust was associated with a broader range of evaluative, social, and control-related judgments, whereas risk showed a more limited association with perceived control. This difference adds nuance to research that frequently considers trust and risk together in explaining digital-service engagement (26, 39).

### Supplementary analyses and interview insights

The supplementary analyses provide additional perspectives on the observed relationships. ANN assigned the highest held-out predictive importance to ATT, SN, and PBC, although its predictive performance did not exceed that of the linear benchmark. NCA identified moderate, statistically supported ceiling relationships for these three TPB constructs, while the six contextual antecedents did not exhibit comparable necessity evidence. These findings complement the structural analysis by examining predictive importance and ceiling patterns as distinct statistical properties.

The extended structural model (M2) further showed that some direct and aggregate indirect associations operated in opposite directions, resulting in relatively small total associations for several contextual antecedents. Nevertheless, the positive associations of ATT, SN, and PBC with CI remained in the extended specification. This pattern draws attention to the importance of distinguishing proximal TPB relationships from the more complex associations involving upstream service conditions.

The interviews provide context for these findings. Participants described both the convenience of digital services and difficulties associated with repeated procedures, uncertainty, and reliance on assistance. Some valued accessible support and offline alternatives while remaining hesitant about completing digital transactions independently. These contrasting experiences help explain why perceptions of inclusive service arrangements and personal control beliefs may follow different patterns.

### Digital inclusion and public health

The findings also have implications for understanding digital inclusion in public-health-related service contexts. As community smart services increasingly provide access to health information, follow-up reminders, and everyday support, digital participation becomes closely connected to opportunities to obtain community resources. Wu et al. (52), in their systematic review of digital exclusion and health among older adults, identified important relationships involving socioeconomic disadvantage, health-related vulnerabilities, contextual barriers, quality of life, and adverse health outcomes.

The present study contributes to this discussion by examining older adults’ continuance intention after initial service adoption. Its findings suggest that continued digital engagement should be considered alongside the institutional arrangements through which services remain accessible. In particular, the different associations of PF and AF with TPB constructs highlight the distinction between confidence in navigating digital procedures and opportunities to obtain services through assistance or alternative channels.

From this perspective, digital inclusion involves both supporting older adults’ capability to use digital services and maintaining equitable opportunities to access essential services when independent digital use is difficult. Understandable procedures, dependable providers, accessible assistance, and non-digital alternatives represent complementary considerations for age-friendly community service provision. This perspective connects the study’s findings on continuance intention with broader discussions of equitable access to community and health-related resources.

### Theoretical contributions

First, this study extends the application of TPB to digitally mediated public services by examining how institutional arrangements and older adults’ digital experiences relate to the three proximal determinants of continuance intention. Integrating service fairness, digital capability and burden, and trust–risk judgments provides a more context-sensitive account of post-adoption intentions in community smart services.

Second, the findings distinguish the associations of different contextual perceptions with TPB judgments. The contrasting patterns of procedural and accessibility fairness highlight the distinction between inclusive service arrangements and perceived control over digital tasks. Similarly, platform trust was associated with a broader range of TPB judgments, whereas perceived risk was primarily associated with perceived control. These differences add conceptual specificity to the roles of fairness, capability, and institutional reliability in continued digital engagement.

Third, the study contributes a post-adoption perspective to digital inclusion research by connecting continuance intention with manageable service procedures, institutional dependability, and inclusive access arrangements. This perspective extends attention beyond the availability of digital channels to the conditions under which older adults perceive continued participation as feasible, with implications for equitable access to community and health-related resources.

### Public health and practice implications

From a public-health perspective, digital inclusion involves both the ability to use digital channels and the opportunity to obtain essential community services, including health-related information and follow-up support. The findings draw attention to two complementary priorities for further evaluation: making digital transactions more manageable and dependable for older users, while maintaining accessible assistance and non-digital alternatives for those who encounter difficulties. These priorities connect individual capability support with inclusive service arrangements.

Trust-related service design could emphasize clear provider identification, predictable responses to errors, and accessible channels for resolving problems.

Digital burden draws attention to repeated data entry, unnecessary verification steps, and confusing navigation. Clear task instructions and ongoing assistance could be evaluated for their contribution to perceived control and successful task completion.

Procedural fairness suggests the value of transparent requirements, consistent online and offline rules, and explanations for rejected requests. Checklists and accessible review channels may help make digital transactions easier to understand; their effects warrant direct service evaluation.

Inclusive provision should also preserve accessible channels, timely assistance, and offline alternatives. These arrangements serve access needs, while support for independent digital completion can be evaluated separately for older users with different capabilities.

### Limitations and future research

The study focused on existing users of community smart services in eight communities across Ningbo, Hangzhou, and Shaoxing. Future research could include non-users, older adults requiring assistance, and communities with different levels of digital-service provision. Baseline records for participants not included in the final analytical sample were unavailable, limiting comparisons of retained and non-retained participants and the assessment of potential attrition-related selection bias.

The three-wave design separated the measurement of contextual antecedents, TPB constructs, and continuance intention, but did not capture changes in the same constructs over time or actual service use. Future studies could combine repeated measurements with service-use records to examine how these relationships develop. The extended model also revealed differences in the directions of some direct and indirect associations, suggesting value in examining alternative model specifications and contextual conditions. Further research could consider family support, technology anxiety, prior service experiences, and reliance on offline channels, while comparing health-related, administrative, and community-participation services.

## Data Availability

The original contributions presented in the study are included in the article/supplementary material, further inquiries can be directed to the corresponding author.
